# Role of Mesenchymal Stem Cells on Cornea Wound Healing Induced by Acute Alkali Burn

**DOI:** 10.1371/journal.pone.0030842

**Published:** 2012-02-17

**Authors:** Lin Yao, Zhan-rong Li, Wen-ru Su, Yong-ping Li, Miao-li Lin, Wen-xin Zhang, Yi Liu, Qian Wan, Dan Liang

**Affiliations:** State Key Laboratory of Ophthalmology, Zhongshan Ophthalmic Center, Sun Yat-Sen University, Guangzhou, People's Republic of China; University of Helsinki, Finland

## Abstract

The aim of this study was to investigate the effects of subconjunctivally administered mesenchymal stem cells (MSCs) on corneal wound healing in the acute stage of an alkali burn. A corneal alkali burn model was generated by placing a piece of 3-mm diameter filter paper soaked in NaOH on the right eye of 48 Sprague-Dawley female rats. 24 rats were administered a subconjunctival injection of a suspension of 2×10^6^ MSCs in 0.1 ml phosphate-buffered saline (PBS) on day 0 and day 3 after the corneal alkali burn. The other 24 rats were administered a subconjunctival injection of an equal amount of PBS as a control. Deficiencies of the corneal epithelium and the area of corneal neovascularization (CNV) were evaluated on days 3 and 7 after the corneal alkali burn. Infiltrated CD68^+^ cells were detected by immunofluorescence staining. The mRNA expression levels of macrophage inflammatory protein-1 alpha (MIP-1α), tumor necrosis factor-alpha (TNF-α), monocyte chemotactic protein-1 (MCP-1) and vascular endothelial growth factor (VEGF) were analyzed using real-time polymerase chain reaction (real-time PCR). In addition, VEGF protein levels were analyzed using an enzyme-linked immunosorbent assay (ELISA). MSCs significantly enhanced the recovery of the corneal epithelium and decreased the CNV area compared with the control group. On day 7, the quantity of infiltrated CD68^+^ cells was significantly lower in the MSC group and the mRNA levels of MIP-1α, TNF-α, and VEGF and the protein levels of VEGF were also down-regulated. However, the expression of MCP-1 was not different between the two groups. Our results suggest that subconjunctival injection of MSCs significantly accelerates corneal wound healing, attenuates inflammation and reduces CNV in alkaline-burned corneas; these effects were found to be related to a reduction of infiltrated CD68^+^ cells and the down-regulation of MIP-1α, TNF-α and VEGF.

## Introduction

Corneal chemical burn is a common ophthalmologic emergency. In general, corneal chemical burn manifests in four phases, including immediate, acute, early repair and late repair phases. Treatment during the acute phase of corneal chemical burn is crucial for its clinical management [1], [2]. In the acute phase of a corneal chemical burn, slow epithelialization, persistent ulceration, corneal perforation and angiogenesis are the most common complications [1], [2]. These complications are closely associated with inflammation, which is an important element during corneal wound healing after a chemical burn [3], [4]. Thus, in the acute phase of a corneal chemical burn, treatments that are anti-inflammatory, anti-angiogenic and that enhance epithelial healing are critical aspects of clinical treatment. A number of treatment modalities have been undertaken to treat corneal chemical burn [1]–[5]. However, no coherent strategy with regard to the ideal treatment of corneal chemical burn yet exists.

Mesenchymal stem cells (MSCs) are a type of multipotent cell originally isolated from bone marrow that have subsequently been isolated from other tissues, such as adipose tissue [6], heart tissue [7], cord blood [8] and oral tissue [9]–[11]. Recently, an increasing body of evidence indicates that MSCs possess multifunctional properties from tissue repair/regeneration to immunomodulatory/anti-inflammatory functions [11]–[14]. More recently, MSCs have been studied for the treatment of corneal chemical burn with encouraging results [15]–[18]. However, for clinical applications, further studies are necessary to confirm and elucidate the therapeutic effects and mechanisms of MSCs in treating corneal chemical burn, especially in the acute phase. In addition, neither application with a special hollow plastic tube nor transplantation with amniotic membrane can be easily carried out in clinical [15], [16]. However, subconjunctival injection is one of the most common clinical administration routes, and it can be easily performed by ophthalmologists clinically.

Thus, this study aimed to investigate the effects and to explore underlying mechanisms of subconjunctival administration of MSCs in the acute phase in a rat corneal alkali burn model.

## Methods

### Animals

All procedures used in this study were in accordance with the principles of the ARVO Statement for the Use of Animals in Ophthalmic and Vision Research. The study was approved by the Research Ethics Committee of the Zhongshan Ophthalmic Center, Sun Yat-sen University (approval ID: 2010-010; Guangzhou, China).

Six-week-old female Sprague-Dawley rats (Guangdong Provincial Center for Animal Experiment, Guangzhou, China) weighing 180–220 g were anesthetized by intraperitoneal injection of 4 ml/kg of 10% chloral hydrate (Zhongshan Ophthalmic Center, Sun Yat-sen University, Guangzhou, China). At the end of the experiment, all rats were sacrificed with an overdose of 10% chloral hydrate. The corneas of the rats were harvested, and only the right eye of each rat was used.

### Isolation, culture and labeling of MSCs

Bone marrow cells were collected by flushing the femurs and tibias of two-week-old female Sprague-Dawley rats with Dulbecco's modified Eagle's medium (DMEM, Gibco-BRL, Grand Island, New York). The cells were cultivated in 75-cm^2^ cell culture flasks in DMEM supplemented with 10% fetal bovine serum (FBS, Gibco-BRL, Grand Island, New York) and penicillin/gentamycin (10 µg/ml, Sigma-Aldrich, St. Louis, MO). After 72 hours, nonadherent cells were removed by changing the culture medium. The culture medium was changed every 3 days, and the cells were transferred when they reached 80% to 90% confluence. Flow cytometry analysis of MSCs and the differentiation of osteoblasts and adipocytes were performed as previously described [19]. MSCs from passages 2–6 were used for all experiments described. On the day of transplantation, MSCs were labeled with DiI (Sigma-Aldrich, St. Louis, MO) and washed twice in phosphate-buffered saline (PBS). The DiI-labeled MSCs were applied to the corneal alkali burn to detect the migration pattern of MSCs in the cornea and conjunctiva by confocal laser scanning microscopy.

### Immunological phenotype of MSCs

For flow cytometry, MSCs from the third passage were trypsinized, resuspended in PBS containing 10% FBS, and incubated with monoclonal antibodies conjugated with either FITC or PE for 30 minutes at 4°C. FITC-conjugated antibodies against CD90 and PE-conjugated antibodies against CD29, CD44, CD34, CD45, and CD11b were purchased from Biolegend (Biolegend, San Diego, CA). Appropriate isotype antibody controls (Biolegend, San Diego, CA) were used in each fluorescence analysis. Cells were analyzed with a BD FACS Calibur, and the data were analyzed using Cell Quest software.

### Differentiation of MSCs in vitro

Both osteogenic and adipogenic differentiation of MSCs were assessed in this study. Briefly, for osteogenic differentiation, MSCs expanded in vitro for 3 passages were cultured for 16 days at 100% confluence in DMEM-high glucose supplemented with 10% FBS, penicillin/gentamycin (10 µg/ml), ascorbic acid (0.2 mM, Sigma-Aldrich, St. Louis, MO), β-glycerophosphate disodium salt pentahydrate (10 mM, Sigma-Aldrich, St. Louis, MO), and dexamethasone (0.1 µM, Sigma-Aldrich, St. Louis, MO). The medium was changed every 3 days. Alizarin red S (2%, Sigma-Aldrich, St. Louis, MO) was used to stain calcium deposits.

To induce adipogenic differentiation, MSCs expanded in vitro for 3 passages were cultured at 100% confluence for 9 days in DMEM-low glucose supplemented with 10% FBS, penicillin/gentamycin (10 µg/ml), indomethacin (0.2 mM, Sigma-Aldrich, St. Louis, MO), insulin (10 µg/ml, Sigma-Aldrich, St. Louis, MO), IBMX (0.5 mM, Sigma-Aldrich, St. Louis, MO), and dexamethasone (1 µM). The medium was changed every 3 days. Oil red O (0.3%, Sigma-Aldrich, St. Louis, MO) was used to stain lipid droplets, which can be easily visualized by light microscopy.

### Animal model of corneal alkali burn

A corneal alkali burn was generated in the right eye of each rat (48 rats). The rats were anesthetized by intraperitoneal injection of 10% chloral hydrate (4 ml/kg). A piece of Whatman #3 filter paper (3-mm diameter) soaked in 4 µl NaOH (1 mol/l) was applied to the center of the cornea for 40 seconds. The cornea was then rinsed with 60 ml of saline for 1 minute.

### Subconjunctival injection of DiI-labeled MSCs

After the corneal alkali burn, the 48 rats were randomly divided into MSC (n = 24) and control (n = 24) groups. Rats in the MSC group received a subconjunctival injection of 0.1 ml PBS containing 2×10^6^ DiI-labeled MSCs immediately and 3 days after the corneal alkali burn. In the control group, rats received a subconjunctival injection of 0.1 ml PBS immediately and 3 days after the corneal alkali burn. All rats in each group were clinically evaluated.

### Observation and examination

Corneal fluorescence staining (8 rats/group) was examined by slit-lamp at 3 and 7 days after the corneal alkali burn (SL-120; Zeiss, Jena, Germany). All observations were performed by a single experienced ophthalmologist who was blind to the allocation of the animals in each group.

To evaluate the development of corneal neovascularization (CNV), ink perfusion via the aorta was performed on days 3 (8 rats/group) and 7 (8 rats/group) as described previously [20]. The eyes were fixed in 10% neutralized buffered formaldehyde overnight, and the corneas were dissected and flattened for image capture. Quantitative measurements of the CNV area were assessed with Image Pro® Plus 5.1 image analysis software (Media Cybernetics, Silver Spring, MD).

### Histological evaluation and migration of MSCs

On day 7 after the corneal alkali burn, 3 eyeballs randomly selected from each group were excised for histological evaluation and the examination of MSCs migration after the rats were sacrificed. Cryosections (6-µm thick) were obtained for hematoxylin-eosin (H-E) and immunofluorescence staining. For immunofluorescence staining, the cryosections were placed in acetone for 15 min at −20°C. The samples were blocked in 1% goat serum albumin (Santa Cruz Biotechnology, Santa Cruz, CA) in PBS for 30 min and subsequently incubated with primary antibody (monoclonal mouse antibody against rat CD68; Santa Cruz Biotechnology, Santa Cruz, CA) diluted in PBS overnight at 4°C. Sections incubated without primary antibody served as negative controls. After washing with PBS, the samples were incubated with a FITC-conjugated secondary IgG antibody for 45 min at room temperature and washed again with PBS. The sections were counterstained with Hoechst (Sigma-Aldrich, St. Louis, MO) for examination by confocal laser scanning microscopy (LSM 510 Meta; Zeiss, Yena, Germany). The numbers of infiltrated cells and CD68+ cells in the corneal stroma were counted in five randomly selected fields (×400) of the H-E and immunofluorescence stained slides by an experienced and independent cell scientist in a blinded condition. Additionally, the slides were monitored for DiI to examine the migration of engrafted MSCs by confocal laser scanning microscopy.

### mRNA expression analysis

The mRNA expression levels of macrophage inflammatory protein-1 alpha (MIP-1α), monocyte chemotactic protein-1 (MCP-1), tumor necrosis factor-alpha (TNF-α) and vascular endothelial growth factor (VEGF) in the corneas were evaluated using real-time polymerase chain reaction (real-time PCR). 7 days after the alkaline burn, 5 corneas from each group were dissected after the rats were sacrificed. Each cornea was cut in half; one half was analyzed using real-time PCR, and the other half was analyzed using an enzyme-linked immunosorbent assay (ELISA). Total RNA was extracted from the harvested corneas with TRIzol (Invitrogen, Carlsbad, CA). The concentration of the total RNA was detected. Genomic DNA was removed by DNase I digestion (Ambion, Austin, TX). Total RNA (1 mg) in a 20 µl reaction volume was reverse transcribed into cDNA using the PrimeScript RT reagent kit (Takara, Dalian, China). The synthesized cDNA was aliquoted. Real-time PCR in 96-well optical plates was performed and analyzed with an ABI PRISM 7000 sequence detection system (Applied Biosystems Inc., Foster City, CA). The relative gene expression levels were calculated according to Oh [15]. The reactions were performed in a 20 µl volume using a SYBR Green reaction mix (Takara, Dalian, China) with 2 µl cDNA. The sequences of the PCR primers are listed in Table 1. The thermal cycling consisted of denaturation for 30 sec at 95°C followed by 40 cycles of 5 sec at 95°C and 30 sec at 60°C. To confirm constant housekeeping gene expression levels in the extracted total RNA, real-time PCR for β-actin was also performed. Real-time PCR was quantified by an SDS 7000 (Applied Biosystems) with rat β-actin as the endogenous control.

**Table 1:** Primer sets for Real-time PCR.

Gene	Forward (5′-3′)	Reverse (5′-3′)
MIP-1α	TCAGCACCATGAAGGTCTCCAC	AGCAAAGGCTGCTGGTCTCAA
MCP-1	CTATGCAGGTCTCTGTCACGCTTC	CAGCCGACTCATTGGGATCA
TNF-α	TCAGTTCCATGGCCCAGAC	GTTGTCTTTGAGATCCATGCCATT
VEGF	GGCTTTACTGCTGTACCTCC	CAAATGCTTTCTCCGCTCT
β-actin	GGAGATTACTGCCCTGGCTCCTA	GACTCATCGTACTCCTGCTTGCTG

### Enzyme-linked immunosorbent assay (ELISA)

VEGF protein levels in the corneas were determined using an ELISA kit (R&D Systems, Minneapolis, MN). The corneas were cut into small pieces, and the tissue was homogenized with a mortar. The samples were lysed in 500 µl of extraction buffer and centrifuged at 10,000 rpm for 10 minutes. The supernatants were used for ELISA. Measurements were conducted according to the instructions of the kit.

### Statistical analysis

The chi-squared test was used to assess the corneal fluorescein staining. The other data are expressed as the mean ± standard deviation. Comparisons of the parameters between the two groups were obtained using Student's t-test for independent samples with SPSS software (SPSS 13.0. Chicago). A p value less than 0.05 was considered statistically significant.

## Results

### Characterization of MSCs

**Figure 1:**
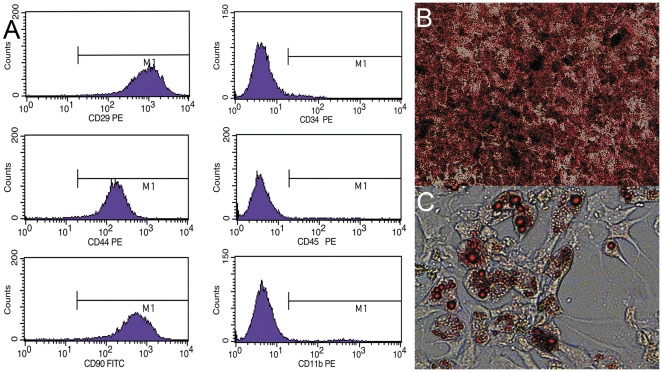
Characterization of MSCs. (A) Flow cytometry analysis of mesenchymal stem cells (MSCs). Expression of CD29, CD44 and CD90 in isolated MSCs. MSCs did not express CD34, CD45 or CD11b. (B) Osteogenic differentiation (Magnification, ×100). (C) adipogenic differentiation (Magnification, ×400).

MSCs were isolated according to their ability to adhere to cell culture plastic. In vitro, these adherent cells were spindle-shaped and proliferated with a well-spread attached morphology. The MSCs expressed high levels of CD29, CD44 and CD90 but did not express the hematopoietic markers CD34, CD45 or CD11b (Fig. 1A). After induction of osteogenic and adipogenic differentiation, alizarin red S and oil red O staining were consistent with osteogenic and adipogenic precursors, respectively (Fig. 1B, 1C).

### Effects of MSCs on epithelial recovery

**Figure 2:**
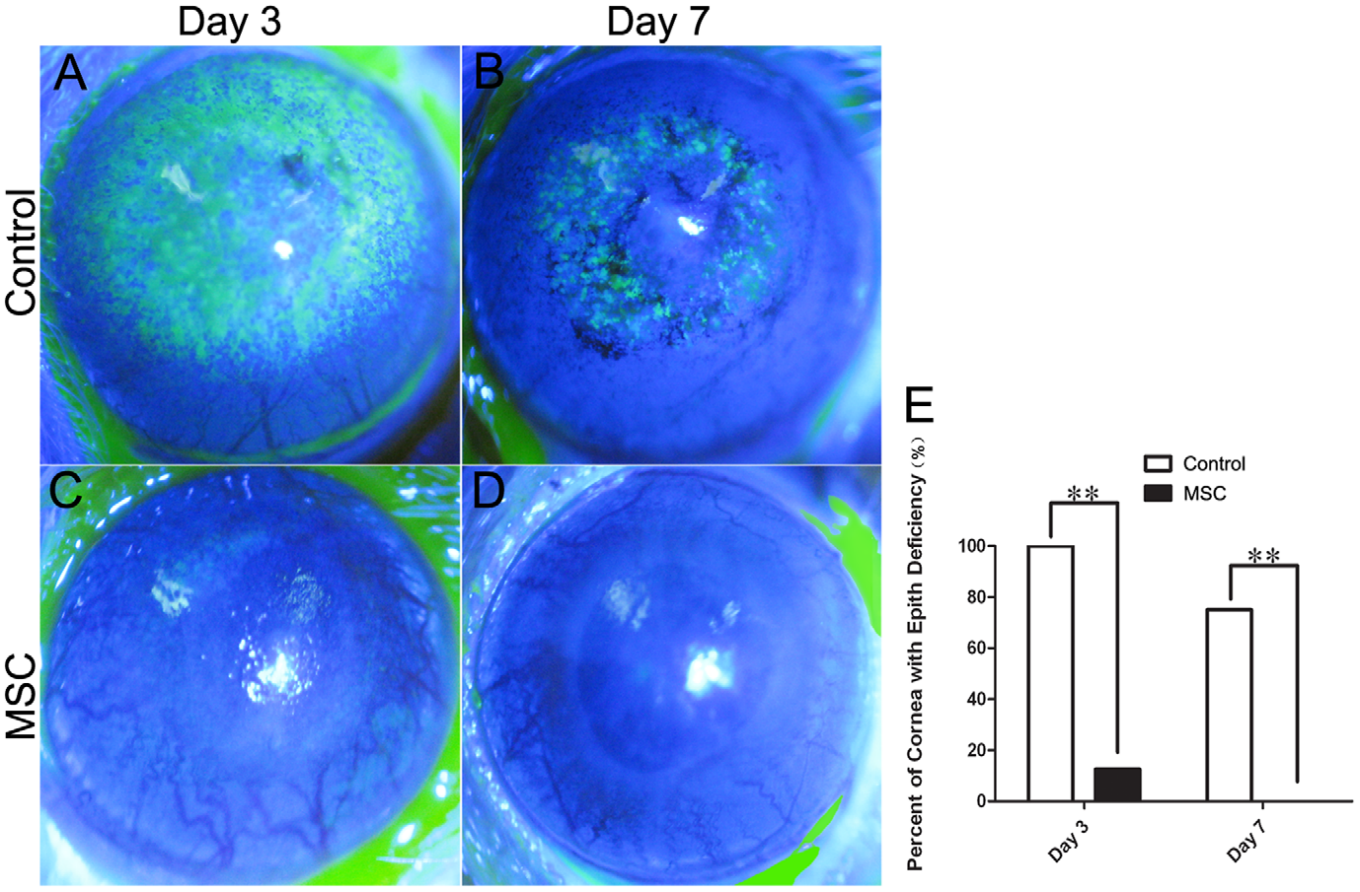
Effects of MSCs on epithelial recovery. (**A–D**) Representative images of fluorescein staining of the cornea on day 3 and day 7 after the alkali burn. Control group on day 3 (**A**) and day 7 (**B**); MSC group on day 3 (**C**) and day 7 (**D**). (**E**) Statistical analysis of number of the fluorescein staining of the cornea on day 3 and day 7. On both day 3 and day 7, the quantity of fluorescein staining of the cornea was significantly lower in the MSC group compared with the control group.

Corneal epithelial staining with fluorescein is indicative of epithelial defects. The recovery of the corneal surface was significantly faster in the MSC group than in the control group. On day 3 after the alkali burn, the corneal epithelium of 7 out of 8 eyes had recovered in the MSC group, whereas all 8 eyes in the control group displayed fluorescent staining consistent with a damaged corneal epithelium (*p* = 0.001; Fig. 2A, 2C, 2E). On day 7, no damaged epithelium was observed in the corneas of the MSC group. However, 6 corneas in the control group exhibited fluorescein staining, consistent with a damaged epithelium (*p* = 0.007; Fig. 2B, 2D, 2E).

### Anti-inflammatory effects of MSCs in the cornea

**Figure 3:**
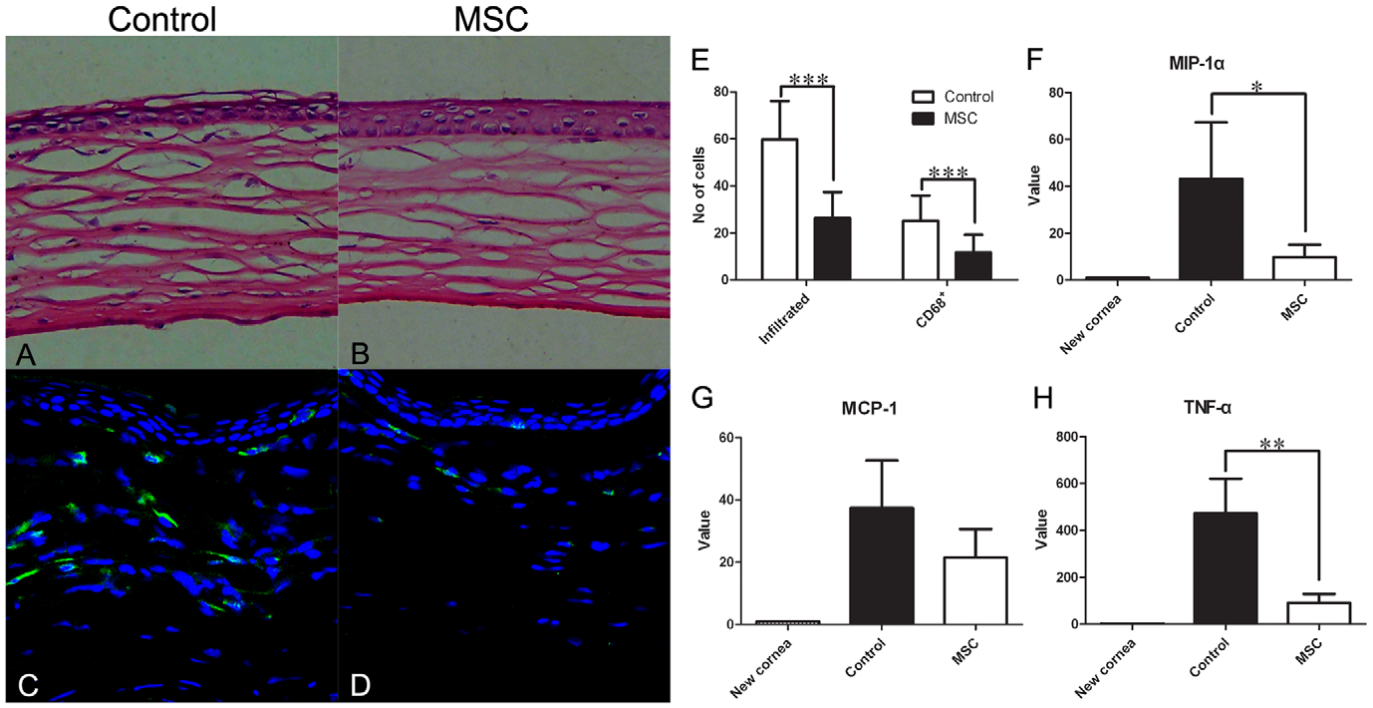
Anti-inflammatory effects of MSCs in the cornea. (**A B**) H-E staining on day 7 (Magnification, ×200); (**C D**) Immunofluorescence staining of CD68 on day 7 (Magnification, ×400); (**A C**) Control group; (**B D**) MSC group. (**E**) Statistical analysis of infiltrated cells and infiltrated CD68^+^ cells in the corneal stroma on day 7 after the corneal alkali burn. (**F–H**) Statistical analysis of real-time PCR results of MIP-1α (**F**), MCP-1 (**G**) and TNF-α (**H**) on day 7 (* *p*<0.05; ** *p*<0.01; *** *p*<0.001).

H-E staining of the eyeball sections revealed that the primary infiltrating cells in the corneal stroma after the corneal alkali burn were inflammatory cells. The numbers of infiltrated cells and CD68^+^ macrophages in the corneal stroma were significantly lower in the MSC group than in the control group (*p* = 0.000 for both; Fig. 3A–E). In the control group, the corneal epithelium was not repaired, and the corneal stroma was thicker and more swollen than that in the MSC group (Fig. 3A, 3B).

To investigate the possible mechanism by which MSCs attenuate inflammation, we assessed the production of the chemotactic factors MIP-1α and MCP-1. We found that the levels of MIP-1α were significantly lower in the MSC group than in the control group (*p* = 0.017; Fig. 3F). However, the expression of MCP-1 was not different between the two groups (*p* = 0.082; Fig. 3G). In addition to MIP-1α, the expression of the immunostimulatory cytokine TNF-α was lower in the MSC group than in the control group (*p* = 0.001; Fig. 3H).

### Effects of MSCs on CNV

**Figure 4:**
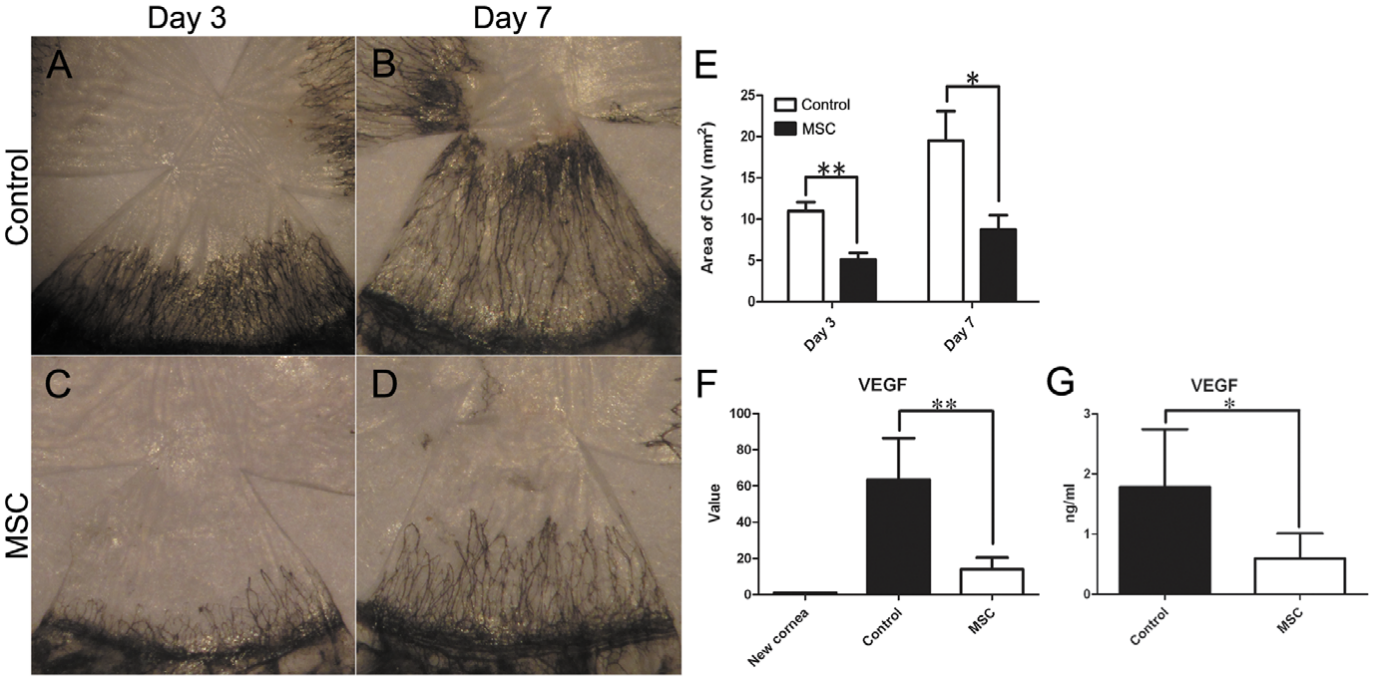
Effects of MSCs on CNV. (**A–D**) Representative images of CNV on day 3 and day 7 after the alkali burn. Control group on day 3 (**A**) and day 7 (**B**); MSC group on day 3 (**C**) and day 7 (**D**). (**E**) Area of CNV on day 3 and day 7 in both groups. (**F G**) Statistical analysis of real-time PCR and ELISA results of VEGF on day 7 (* *p*<0.05; ** *p*<0.01).

On days 3 and 7 after the corneal alkali burn, CNV occurred in both the control and MSC groups (Fig. 4A–D). Compared to the corneas of the control group, corneas in the MSC group showed less CNV. The amount of CNV was quantified by measuring the area of neovascularization. We found that the area of CNV was significantly smaller in the MSC group than in the control group (*p* = 0.001 on day 3, *p* = 0.016 on day 7; Fig. 4E), suggesting an inhibitory effect of MSCs on CNV.

We further investigated the expression of VEGF to determine the possible effect of MSCs on corneal angiogenesis. Both the mRNA and protein levels of VEGF were significantly lower in the MSC group compared to the control group (*p* = 0.002 and *p* = 0.035, respectively; Fig. 4F, 4G), indicating a decrease in corneal angiogenesis caused by MSCs.

### Migration of MSCs

**Figure 5:**
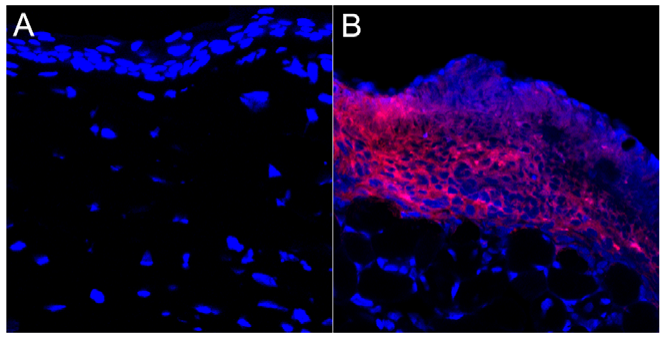
Engraftment of MSCs. (**A**) No DiI-labeled MSCs were detected in the cornea on day 7 after the corneal alkali burn. (**B**) A large number of DiI-labeled MSCs were detected in the subconjunctival tissues on day 7 after the corneal alkali burn (Magnification, ×400).

To investigate whether the DiI-labeled MSCs were successfully engrafted after the corneal alkali burn, we traced the migration of MSCs in the host by labeling them with DiI and detecting them via confocal laser scanning microscopy. A large number of DiI-labeled MSCs remained subconjunctival as they were originally engrafted; no MSCs had infiltrated into the cornea (Fig. 5A, 5B).

## Discussion

Corneal chemical burn is a common type of ocular injury. It often results in extensive damage and permanent visual impairment in severe cases and in cases lacking proper management. Therefore, prompt and appropriate treatment is necessary, especially in the acute phase of a corneal chemical burn. Recently, MSCs, a type of multipotent cell, have become a promising approach for the treatment of corneal chemical burn [15]–[18]. Studies have shown that MSCs treatment enhances wound healing and reconstitutes the corneal surface due to MSCs differentiation [17], [18]. However, others have demonstrated that the MSCs may exert their beneficial effects by inhibiting inflammation and angiogenesis rather than by differentiation [15], [16]. Thus, further studies are needed to elucidate the mechanisms utilized or influenced by MSCs. In our study, consistent with previous studies, we showed that subconjunctival injection of MSCs accelerated corneal wound healing [15], [16]. Importantly, we found that a large number of DiI-labeled MSCs remained subconjunctival as they had originally been engrafted, and none of them infiltrated into the injured cornea (Figure 5A, B). Our results also showed that subconjunctival injection of MSCs attenuated the inflammation of the locally burned cornea by inhibiting inflammatory cell infiltration and proinflammatory cytokine production. These results further support previous studies [15], [16], and they suggest that MSCs treatment can accelerate the wound healing of a chemically burned cornea via anti-inflammatory and anti-angiogenic activity. In addition, some studies have shown beneficial effects of MSCs in the treatment of corneal chemical burn in the early repair phase [16], [18]. However, for corneal chemical burn, treatment in the acute phase is crucial. Thus, this study focused on the therapeutic effects of MSCs in the acute phase of corneal chemical burn.

The studies of Ma et al. [16] and Oh et al. [15] reported that MSCs treatment enhanced wound healing and accelerated corneal surface reconstruction via its anti-inflammatory effects. However, the anti-inflammatory mechanism of MSCs in corneal chemical burns remains elusive. The study of Ma et al. [16] speculated that the therapeutic anti-inflammatory effects of MSCs may be associated with their inhibition of the expression of CD45, IL-2 and MMP2. The study of Oh et al. [15] further indicated that MSCs exert their anti-inflammatory effects by suppressing the infiltration of adaptive CD4^+^ T cells and the expression of CD4^+^ T cell-related cytokines (IL-2, IFN-γ) and MMP2 possibly by secreting soluble factors. However, in corneal chemical burn, when the cornea is injured, corneal epithelial cells and other local cells immediately release a large quantity of mediators, including inflammatory mediators and chemotactic factors, to recruit inflammatory cells into the injured cornea. At 12–24 hours after the original injury, innate immune cells (such as neutrophils and macrophages) arrive at the cornea and release inflammatory mediators that contribute to the development of corneal disorders [21]. Therefore, inhibiting the early inflammatory reaction of corneal cells and innate immune cells is critical for the early treatment of corneal chemical burn. In our study, we first showed that MSCs treatment suppresses the infiltration of inflammatory cells and CD68^+^ macrophages, which are closely related to the degree of inflammation [22]. Next, we found that MSCs may inhibit macrophage infiltration maybe by suppressing the expression of the macrophage chemokine MIP-1α [23]. In addition, previous studies have demonstrated that MSCs are capable of inhibiting proinflammatory cytokine (TNF-α) production by macrophages [24], [25]. Thus, we also examined whether MSCs treatment is capable of inhibiting TNF-α production in a locally burned cornea. Our results showed that subconjunctival MSCs significantly decrease the production of TNF-α in locally burned cornea. These findings, combined with the aforementioned pathological progress of corneal chemical burn, indicate that the anti-inflammatory mechanism of MSCs in the acute phase may be that they first inhibit the release of MIP-1α by local corneal cells and that subsequently, they decrease the infiltration of macrophages and TNF-α production.

Currently, it is thought that MSCs provide therapeutic effects through both cell-membrane contact and soluble factors [26]–[28]. In this study, we found that few MSCs could infiltrate into the wounded cornea. However, on day 7 after the corneal alkali burn, a large number of DiI-labeled MSCs had accumulated in the subconjunctival tissue where the MSCs were injected. Therefore, we speculate that soluble factors secreted by the MSCs rather than cell-membrane contact played an important role in this model.

Limbal stem cell (LSC) transplantation has been widely used clinically for ocular surface reconstruction in corneal chemical burns. However, it is generally accepted that LSC transplantation should be used in the early or late repair phase of a corneal chemical burn rather than in the acute phase [29]–[31]. The current treatment options for corneal chemical burn are prompt anti-inflammatory therapy in the early phase and the provision of LSC in the late stage after the inflammation has subsided [32], [33]. Unlike LSC transplantation, in our study, MSCs treatment could be applied repeatedly in the acute phase of a corneal chemical burn. It is also expected that in future clinical settings, MSCs will be used first to inhibit inflammation and angiogenesis, followed by LSC transplantation to reconstruct the ocular surface.

In addition, LSC transplantation has its limitations in clinical applications. First, for autotransplantation, it is not suitable for binoculus sufferers because it risks damaging the comparatively healthy eye. Second, for allotransplantation, the risk of significant side effects from long-term immunosuppression is a major drawback. Unlike LSC, MSCs are easy to isolate in sufficient numbers for clinical autotransplantation or allotransplantation. Moreover, our study and other studies have shown encouraging results of MSCs allotransplantation in both the acute phase (inhibiting inflammation and angiogenesis) and the early repair phase (reconstructing ocular surface) of corneal chemical burn [15]–[18]. Taken together, these findings show the great superiority of the MSCs application in the clinical setting.

To administer MSCs to the injured cornea of rats, previous studies have used intravenous infusion, amniotic membrane as a carrier or a special hollow plastic tube [15]–[18]. However, for clinical applications, these methods are difficult to perform and are not accepted by ophthalmologists for treating corneal disorders. Unlike these methods, subconjunctival injection is a common and easy clinical administration route for the treatment of ophthalmological disorders. Thus, in this study, we used subconjunctival injection as a route to administer MSCs. Consistent with previous studies [15], [16], our study showed similartherapeutic effects on corneal chemical burn by subconjunctival injection of MSCs. This finding suggests that subconjuctival injection is a good alternative for the clinical application of MSCs to corneal chemical burn.

In summary, our study demonstrated that topical subconjunctival injection of MSCs accelerates corneal epithelial recovery and inhibits neovascularization in a corneal alkali burn rat model. Mechanistically, our results found that MSCs may exert their therapeutic effects by suppressing the early inflammatory reaction of local corneal cells and innate immune cells via paracrine mechanisms. These findings further confirm and elucidate the anti-inflammatory mechanism of MSCs in the acute phase of a corneal chemical burn, and they suggest that the subconjunctival administration of MSCs could be a good alternative treatment for the clinical management of corneal chemical burn.

## References

[1] Wagoner MD (1997). Chemical injuries of the eye: current concepts in pathophysiology and therapy.. Surv Ophthalmol.

[2] Brodovsky SC, McCarty CA, Snibson G, Loughnan M, Sullivan L (2000). Management of alkali burns: an 11-year retrospective review.. Ophthalmology.

[3] Kuo IC (2004). Corneal wound healing.. Curr Opin Ophthalmol.

[4] Adamis AP, Aiello LP, D'Amato RA (1999). Angiogenesis and ophthalmic disease.. Angiogenesis.

[5] He J, Bazan NG, Bazan HE (2006). Alkali-induced corneal stromal melting prevention by a novel platelet-activating factor receptor antagonist.. Arch Ophthalmol.

[6] Zannettino AC, Paton S, Arthur A, Khor F, Itescu S (2008). Multipotential human adipose-derived stromal stem cells exhibit a perivascular phenotype in vitro and in vivo.. J Cell Physiol.

[7] Hoogduijin MJ, Crop MJ, Peeters AM, Van Osch GJ, Balk AH (2007). Human heart, spleen, and perirenal fat-derived mesenchymal stem cells have immunomodulatory capacities.. Stem Cells Dev.

[8] Oh W, Kim DS, Yang YS, Lee JK (2008). Immunological properties of umbilical cord blood-derived mesenchymal stromal cells.. Cell Immunol.

[9] Zhang Q, Shi S, Liu Y, Uyanne J, Shi Y (2009). Mesenchymal stem cells derived from human gingiva are capable of immunomodulatory functions and ameliorate inflammation-related tissue destruction in experimental colitis.. J Immunol.

[10] Zhang QZ, Su WR, Shi SH, Wilder-Smith P, Xiang AP (2010). Human gingiva-derived mesenchymal stem cells elicit polarization of m2 macrophages and enhance cutaneous wound healing.. Stem Cells.

[11] Su WR, Zhang QZ, Shi SH, Nguyen AL, Le AD (2011). Human gingiva-derived mesenchymal stromal cells attenuate contact hypersensitivity via prostaglandin E(2) -dependent mechanisms.. Stem Cells.

[12] Nauta AJ, Fibbe WE (2007). Immunomodulatory properties of mesenchymal stromal cells.. Blood.

[13] Zhao S, Wehner R, Bornhauser M, Wassmuth R, Bachmann M (2010). Immunomodulatory properties of mesenchymal stromal cells and their therapeutic consequences for immune-mediated disorders.. Stem Cells Dev.

[14] Uccelli A, Moretta L, Pistoia V (2008). Mesenchymal stem cells in health and disease.. Nat Rev Immunol.

[15] Oh JY, Kim MK, Shin MS, Lee HJ, Ko JH (2008). The anti-inflammatory and anti-angiogenic role of mesenchymal stem cells in corneal wound healing following chemical injury.. Stem Cell.

[16] Ma Y, Xu Y, Xiao Z, Yang W, Zhang C (2005). Reconstruction of chemically burned rat corneal surface by bone marrow-derived human mesenchymal stem cells.. Stem Cells.

[17] Ye J, Yao K, Kim JC (2006). Mesenchymal stem cell transplantation in a rabbit corneal alkali burn model: engraftment and involvement in wound healing.. Eye (Lond).

[18] Jiang TS, Cai L, Ji WY, Hui YN, Wang YS (2010). Reconstruction of the corneal epithelium with induced marrow mesenchymal stem cells in rats.. Mol Vis.

[19] Popp FC, Slowik P, Eggenhofer E, Renner P, Lang SA (2007). No contribution of multipotent mesenchymal stromal cells to liver regeneration in a rat model of prolonged hepatic injury.. Stem Cells.

[20] Su W, Li Z, Lin M, Li Y, He Z (2011). The effect of doxycycline temperature-sensitive hydrogel on inhibiting the corneal neovascularization induced by BFGF in rats.. Graefes Arch Clin Exp Ophthalmol.

[21] Wilson SE, Mohan RR, Mohan RR, Ambrosio R, Hong J (2001). The corneal wound healing response: cytokine-mediated interaction of the epithelium, stroma, and inflammatory cells.. Prog Retin Eye Res.

[22] Choi JA, Choi JS, Joo CK (2011). Effects of amniotic membrane suspension in the rat alkali burn model.. Mol Vis.

[23] DiPietro LA, Burdick M, Low QE, Kunkel SL, Strieter RM (1998). MIP-1alpha as a critical macrophage chemoattractant in murine wound repair.. J Clin Invest.

[24] Maggini J, Mirkin G, Bognanni I, Holmberg J, Piazzón IM (2010). Mouse bone marrow-derived mesenchymal stromal cells turn activated macrophages into a regulatory-like profile.. PLoS One.

[25] Aggarwal S, Pittenger MF (2005). Human mesenchymal stem cells modulate allogeneic immune cell responses.. Blood.

[26] Lee JW, Fang X, Krasnodembskaya A, Howard JP, Matthay MA (2011). Mesenchymal stem cells for acute lung injury: role of paracrine soluble factors.. Stem Cells.

[27] Krampera M, Glennie S, Dyson J, Scott D, Laylor R (2003). Bone marrow mesenchymal stem cells inhibit the response of naive and memory antigen-specific T cells to their cognate peptide.. Blood.

[28] Groh ME, Maitra B, Szekely E, Koc ON (2005). Human mesenchymal stem cells require monocyte-mediated activation to suppress alloreactive T cells.. Exp Hematol.

[29] Shi W, Wang T, Gao H, Xie L (2009). Management of severe ocular burns with symblepharon.. Graefes Arch Clin Exp Ophthalmol.

[30] Fogla R, Padmanabhan P (2005). Deep anterior lamellar keratoplasty combined with autologous limbal stem cell transplantation in unilateral severe chemical injury.. Cornea.

[31] Borderie V, Touzeau O, Bourcier T, Allouch C, Scheer S (2003). Treatment of the sequelae of ocular burns using limbal transplantation.. J Fr Ophtalmol.

[32] Kuckelkorn R, Schrage N, Keller G, Redbrake C (2002). Emergency treatment of chemical and thermal eye burns.. Acta Ophthalmol Scand.

[33] Fish R, Davidson RS (2010). Management of ocular thermal and chemical injuries, including amniotic membrane therapy.. Curr Opin Ophthalmol.

